# Reciprocal facilitation between large herbivores and ants in a semi-arid grassland

**DOI:** 10.1098/rspb.2018.1665

**Published:** 2018-10-10

**Authors:** Xiaofei Li, Zhiwei Zhong, Dirk Sanders, Christian Smit, Deli Wang, Petri Nummi, Yu Zhu, Ling Wang, Hui Zhu, Nazim Hassan

**Affiliations:** 1 Institute of Grassland Science/School of Environment, Northeast Normal University, and Key Laboratory of Vegetation Ecology/Key Laboratory for Wetland Ecology and Vegetation Restoration, Changchun, Jilin 130024, People's Republic of China; 2 Environment and Sustainability Institute, University of Exeter, Penryn Campus, Penryn, Cornwall TR10 9FE, UK; 3 Conservation Ecology Group, Groningen Institute for Evolutionary Life Sciences, University of Groningen, PO Box 11103, 9700, CC, Groningen, The Netherlands; 4 Wetland Ecology Group, Department of Forest Sciences, University of Helsinki, PO Box 27, 00014 University of Helsinki, Finland

**Keywords:** facultative mutualism, ecosystem engineering, facilitation, resources availability, indirect effects, soil nutrients

## Abstract

While positive interactions have been well documented in plant and sessile benthic marine communities, their role in structuring mobile animal communities and underlying mechanisms has been less explored. Using field removal experiments, we demonstrated that a large vertebrate herbivore (cattle; *Bos tarurs*) and a much smaller invertebrate (ants; *Lasius* spp.), the two dominant animal taxa in a semi-arid grassland in Northeast China, facilitate each other. Cattle grazing led to higher ant mound abundance compared with ungrazed sites, while the presence of ant mounds increased the foraging of cattle during the peak of the growing season. Mechanistically, these reciprocal positive effects were driven by habitat amelioration and resource (food) enhancement by cattle and ants (respectively). Cattle facilitated ants, probably by decreasing plant litter accumulation by herbivory and trampling, allowing more light to reach the soil surface leading to microclimatic conditions that favour ants. Ants facilitated cattle probably by increasing soil nutrients via bioturbation, increasing food (plant) biomass and quality (nitrogen content) for cattle. Our study demonstrates reciprocal facilitative interactions between two animal species from phylogenetically very distant taxa. Such reciprocal positive interactions may be more common in animal communities than so far assumed, and they should receive more attention to improve our understanding of species coexistence and animal community assembly.

## 1. Introduction

The last two decades has seen increasing interest in the role of facilitation in structuring ecological communities [1–7], with facilitation defined as any interaction that benefits at least one of the participants and causes net harm to neither [8]. Several attempts have been made to place facilitation into broader ecological theory [8–10], particularly with the stress gradient hypothesis [1,11,12].

While facilitation has been well documented in plant and sessile (or less mobile) communities [2,4,7,13–17], its importance in structuring more mobile animal communities has been less explored. Evidence is growing that facilitation between animal species may be common and can have far-reaching consequences for species abundance, distribution and diversity in ecosystems [18–24]. Still, the difficulty in elucidating the operating mechanisms behind the patterns may hinder study of facilitative interactions in animal communities. Mobile animal species are often separated in space and time, making their interspecific interactions difficult to detect and document [22].

Several mechanisms have been proposed to explain facilitative interactions in animal communities. First, one species can benefit another by improving accessibility to, or quality of, resources. A classic example is large herbivore grazing that induces ‘compensatory regrowth’ in plants, resulting in enhanced forage quality (biomass and nitrogen (N) content) of grasses that benefits other grazers in Africa savannahs [21,25]. Second, one species can benefit another by ameliorating abiotic conditions in particular habitats. A classic example of this are beavers (*Castor fiber*) in riparian ecosystems, that act as ‘ecosystem engineers’ [26,27] by their dam-building activities that lead to the formation of extensive wetland habitats, which enhances the abundance and diversity of other animals such as butterflies, waterbirds and bats [23,28–30]. Third, a species may facilitate another by modifying the behaviour or population dynamics of predators [31] or competitors [32,33]. However, despite these examples, few studies on facilitation in animal communities have been able to pinpoint the underlying mechanisms, because many of the interactions are cryptic and complex, and often involve various trophic levels, habitat structure and a behaviour component. Hence, understanding the actual mechanisms behind animal facilitation remains a challenge.

To date, the majority of animal facilitation studies have focused at unidirectional effects, in particular between species that are very different in body size, often in the form of animal species benefitting the smaller ones [20,34–36]. However, small animal species—often high in abundance or biomass—have the potential to feedback on large animal species as well [37–41]. For example, the bioturbating activities of soil fauna such as termites, earthworms and dung beetles help to aerate and fertilize the soil and so improve the quality of the forage for large grazers [38–44]. So far, the reciprocal facilitative interactions between large and small animal species, often from very different taxa, have received little attention. Yet these reciprocal facilitative interactions may be much more common than assumed so far, importantly explaining spatial patterns observed at the landscape scale [41,44]. Hence, it is time to think outside the (taxonomic) box and consider reciprocal facilitative interactions between dissimilar species [45].

In this study, we examine the potential reciprocal facilitative interactions between two phylogenetic distant taxa, namely cattle (*Bos taurus*) and ants (*Lasius* spp.). In our study system, cattle are the dominant aboveground vertebrates, while ants are the dominant invertebrate insects belowground, with *Lasius alienus* and *Lasius flavus* accounting for greater than 60% of all ant individuals [46]. *Lasius* spp. ants prefer a dry, sunny microclimate and generally avoid habitats with thick vegetation and/or ground litter layer [46–48]. Large vertebrate herbivores reduce vegetation biomass as well as plant litter accumulation, both by their direct consumption of plant tissues and indirect effects of trampling that accelerate litter decomposition processes [21]. Thereby, cattle control the habitat characteristics created by plants and litter and this could potentially benefit ants. Conversely, activities of ants, especially those of *Lasius* spp., are known to enhance soil nutrient availability and change soil moisture [48,49]. Such changes in soil conditions can increase vegetation growth [50,51], which may in turn facilitate aboveground herbivore consumers [39,52].

**Figure 1.**
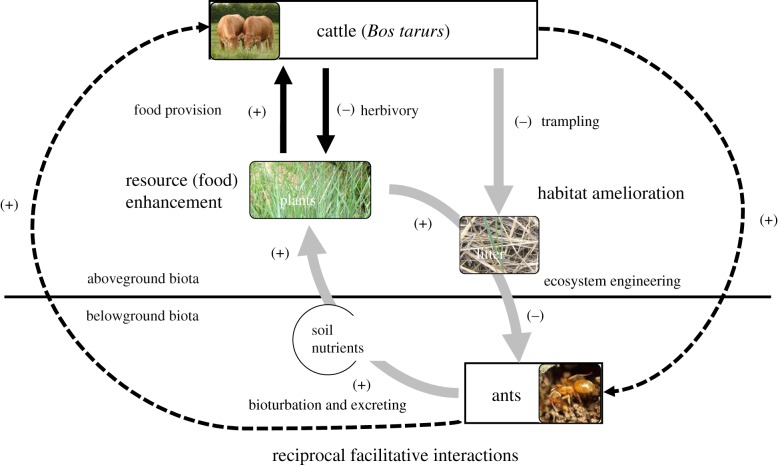
The hypothesized mechanisms for mutualistic interactions between cattle (*Bos tarurs*) aboveground and ants belowground mediated by trophic and non-trophic effects in a semi-arid grassland in northeastern China. Trophic effects (e.g. herbivory) are shown by black arrows, non-trophic effects (e.g. ecosystem engineering) by grey arrows. The facilitative effects of cattle on ants and vice versa are denoted by dashed black lines. Plus sign in brackets indicates positive effects, while the minus sign in brackets indicates negative effects.

We test the general hypothesis that cattle and ants can exert reciprocal, facilitative effects on each other by habitat amelioration and resource (food) enhancement. More specifically, we expect that grazing and trampling by cattle will reduce vegetation and litter biomass and so create more open micro-habitats that favour ants. By their turn, bioturbation (e.g. mound building) activities of ants will enhance soil nutrient availability that increases plant (food) quantity and/or quality and so benefit cattle (figure 1). To test these hypotheses, we explored the responses of ant (mound) abundance and cattle feeding behaviours in a manipulated animal removal field-experiment. To reveal the potential underlying mechanisms, we assessed how cattle and ant manipulations altered soil nutrients, plant quantity and quality, and plant and litter cover.

## 2. Study site and methods

### (a) Study system and background

The study was conducted in a semi-arid low elevation (approx. 150 m) grassland in the Jilin Province of Northeast China (44°45′ N, 123°45′ E). Annual mean temperature ranges from 4.6 to 6.4°C and annual precipitation is 280–400 mm. The area is dominated by the perennial grass *Leymus chinensis*. Other plants include the grasses *Phragmites australis* and *Calamagrostis epigejos*, as well as the forbs *Artemisia scoparia* and *Kalimeris integrifolia* [53]. The soil is a mixed salt-alkali meadow steppe (Salid Aridisol, US Soil Taxonomy) of 29% sand, 40% silt and 31% clay (top 10 cm) and is nutrient-poor with total N content ranging from 2.2 to 2.5 mg g^−1^, and total phosphorus (P) content ranging from 0.23 to 0.27 mg g^−1^ [54]. The area has a long-standing tradition of low-intensity livestock grazing with cattle and sheep, as well as mowing for hay making. Natural vertebrate herbivores such as geese and rodents are rare in the area. Furthermore, the area hosts a density (*ca* 0.1–0.5 mounds every 1 m^2^) of nests of the yellow ants, *La. alienus* and *La. flavus*, with an average mound height of 7.0 (s.e. 0.5) cm and a mean mound base diameter of 40 (s.e. 3.4) cm (X. Li, Z. Zhong, D. Wang, Y. Zhu, H. Zhu, L. Wang, N. Hassan 2018, unpublished data).

### (b) Experimental set-up

The study area was fenced in 2005 to protect against uncontrolled human disturbance (e.g. grazing and mowing). In June 2009, we established twelve 50×50 m enclosure plots with the treatment factor ‘cattle grazing’ at the plot level and ‘ant presence’ at a subplot level arranged in a randomized block design, i.e. with six blocks each containing a pair of experimental plots (electronic supplementary material, figure S1). Distance between experimental blocks was 150–300 m, and the distance between plots in a block was on average 30 m. Each enclosure plot was divided into eight 3 × 3 m randomly located subplots, separated by ±7 m. For the two plots within each block, we randomly applied one 50 × 50 m plot to cattle grazing, while the other served as a control (ungrazed) plot. For the eight 3 × 3 m subplots within each plot, we randomly assigned four of them to the ant suppression treatment (ant suppressed), while the other four were left unmanipulated as control treatments (ant present) (electronic supplementary material, figure S1). Thus, we had four experimental treatments in a fully crossed 2 × 2 nested design, i.e. cattle only (C), cattle + ants (C + A), ants only (A), and no cattle and no ants (None).

#### (i) Grazing treatment

From 2010 to 2013, the plots were grazed by cattle (mean weight 300 ± 8 kg, mean ± s.e.) at an equal light to moderate intensity (about 30% of aboveground plant biomass consumed by cattle), a recommended grazing intensity by local governments. A total of 48 mature cattle were assigned to the six grazed plots, with eight cattle heads per grazed plot. Grazing occurred each year from June to September during the first two weeks of each month, with a daily grazing regime between 06.00–08.00 h and 16.00–18.00 h, creating grazing intensities similar with local grazing habit.

#### (ii) Ant suppression treatment

From 2010 to 2013, we applied 10 g of poison ant baits (Jingkang Ant Bait Granules, Lekang Technology, Beijing, China) around the entrance of active ant nests to suppress ants in the ant suppression subplots from June to August, the active period of ants in each year. The main active ingredients of the ant bait are 0.45% Tetramethrin and 0.02% Alpha-cypermethrin. The ant bait is specifically designed to appeal to ants and kill their colonies and has been used successfully in reducing ant populations in the region. Additional experiments indicate that, except for ants (and crickets, see electronic supplementary material, figure S4), the ant bait has limited impacts on other arthropods, plant growth, soil nutrients and cattle behaviours in our system (see the electronic supplementary material, figure S4–S6). We did not install barriers to prevent ants from recolonizing the subplots (as did Wardle *et al*. [55]), because it would exert a significant physical disturbance to soil and vegetation, and alter the cattle feeding behaviours (based on our field pre-trials). Instead, to minimize the potential biases, we considered the outermost 1 m of each 3 × 3 m ant-manipulation subplot as a ‘buffer’ and avoided sampling in these areas. Our ant suppression treatments dramatically dropped total active ant nest densities (see Results below).

### (c) Initial conditions

In August (peak of the growing season) 2009, 1 year before the beginning of cattle grazing and ant suppression treatments, we measured the initial conditions, including plant community characteristics, soil properties, microclimate and ant abundance, within the eight 3 × 3 m subplots in each plot.

We measured biomass of each plant group (the dominant *Le. chinensis* grasses, other grasses and forbs), total plant biomass, plant litter biomass and plant nutrient content. We estimated aboveground plant biomass by clipping plants to ground level in 1 × 0.2 m area in two random locations within each of the eight subplots. The aboveground biomass was sorted into *Le. chinensis*, ‘other grasses’, and ‘forbs’. In addition, we collected plant litter in the same locations. Aboveground biomass and litter were then dried for 48 h at 70°C and weighed. We measured the N content of the three plant groups using an automatic Kjeldahl nitrogen analyzer (Kjeltec^®^ 2300 Analyzer Unit, Foss Analytical AB, Höganäs, Sweden), after we ground the dried plant samples of each group (*Le. chinensis*, other grasses, and forbs) through a 0.8 mm mesh screen in a Wiley mill.

For soil properties, soil moisture was determined using a handheld soil moisture reader (OSA-1, OUSU Technology, Hebei, China), taking readings from five random locations within each of the eight subplots. Soil nutrients were determined by using a 4 cm diameter soil auger to randomly collected five replicate 0–20 cm soil samples from each subplot, which were pooled to homogenize the samples. For each soil sample, a 10 g subsample was extracted with 70 ml 2 mol l^−1^ KCl. Extracts were frozen at 20°C for analysis of 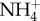 and 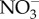 content by continuous flow analyser (Alliance Flow Analyzer; Futura, Frépillon, France). Total soil N was the sum of 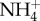 and 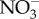 concentrations. For soil total available P, another 10 g subsample soil was extracted using acidified NH4OAc-EDTA and analysed by ICP (Spectro Analytical Instruments, Marlborough, MA, USA).

We measured light penetration, air temperature and humidity at the soil surface by taking readings from two random locations within each subplot. Light penetration was measured using a GLZ-C-G PAR (photosynthetically active radiation) point sensor (Top Instrument, Zhejiang, China), taking light intensity readings from above the vegetation canopy and from the ground surface. We measured ambient air temperature and relative humidity using an AR-847 digital thermo-hygrometer (Jinzhan Inc., Shenzhen, China).

We visually assessed the total number of active ant nests and the number of active *Lasius* ant nests in the subplots. *Lasius* ants make typical aboveground mounds and are relatively easy to identify. We checked whether the ant nests were active by visually examining if there was any ‘fresh’ soil deposited around the entrance of the mound, and by inserting a 30 cm plastic wire into the mounds for 10 s to see if any ants would come out.

### (d) Effects of cattle grazing on ants, plants, litter, and microclimate

In August 2012, we investigated the effects of 3 year (2010–2012) cattle grazing on ant nest density in the four 3 × 3 m ant-present subplots in the six grazed and the six ungrazed plots using the same methodologies as described above. Ant nest density was assessed on 14 August and 30 August in 2012. We averaged the ant nest data for each plot over time (two sampling dates for each subplot) and across the four ant-present subplots in each plot and used this one data point per plot in the statistical analyses. On 25 August 2012, to investigate the mechanisms by which cattle grazing could affect ant nest density, we measured plant biomass, litter biomass and microclimate (light penetration, air temperature and air relative humidity) using the same methods as above.

### (e) Effects of ants on cattle feeding behaviour, plants and soils

On 5 August and 12 August, we recorded the total number of visits and total grazing time (recorded and calculated to the second) by cattle in the subplots. We considered a cattle-visit when there was at least one leg into the subplots for more than 3 s, and considered a cattle grazing activity as when an animal was feeding on plants in the subplots for more than 3 s. The observations were conducted twice daily (from 06.00 to 08.00 h and from 16.00 to 18.00 h). We averaged the feeding behaviour data from the two sampling dates for each subplot, then we averaged the feeding behaviour data from the four ant suppression and the four ant-present 3 × 3 m subplots in each cattle grazed plot and used these data in the statistical analyses.

On 27 August 2012, to investigate the mechanisms by which ants could affect cattle feeding behaviour, we measured living plant biomass of each plant group (*Le. chinensis*, other grasses and forbs), total plant biomass, and plant N contents of each plant group, and soil moisture and soil nutrients, such as soil total available N and P in the four ant suppression and the four ant-present subplots within each cattle grazed plot using the methodology described above. We averaged plant and soil condition data for the four ant suppression and the four ant-present 3 × 3 m subplots in each cattle grazed plot for statistical analyses.

### (f) Additional plant-litter-removal experiment

In 2012, we conducted an additional plant-litter-removal experiment to further investigate the influence of plant litter on ant nest density, independent of cattle grazing. In May 2012, six pairs of 3 × 3 m plots were randomly placed in the field outside the grazing areas. We randomly selected one plot of each pair and removed plant litter on the soil surface, while the other plot served as the control. We repeated the experimental treatments in the plots in 2013. In mid-August 2013, we measured *Lasius* ant nest density and total ant nest density, by visually counting the number of active *Lasius* ant nests and total ant nests in the plots, respectively.

### (g) Data analyses

For all variables discussed above, we averaged each variable for the four replicate 3 × 3 m subplots within each grazed and control plot for statistical analyses. All data were assessed for normality and analysed using the open source software R 3.1.0 [56]. We used linear mixed effects models from the nlme package [57] to test for the effects of cattle grazing on ants, plants, litter and microclimate. Ant nest density, plant biomass, litter biomass and microclimate were included as response variables, while cattle grazing treatment (two levels: grazed and ungrazed) was included as a fixed factor and block as a random factor. We then tested for relationships between plant litter biomass and total active ant nest density in all the plots with a linear model. The effects of plant litter (two levels: litter present and removed) on ants in the plant-litter-removal experiment were analysed using linear models based on generalized least squares. This was necessary to account for unequal variances for the treatment groups. We used VarIdent to account for variance heterogeneity in effect sizes between treatment groups. We further analysed the impact of ant nest presence on cattle behaviours with total number of cattle visits and total grazing time in the 3 × 3 m subplots in the six cattle grazed plots as the response variable using linear mixed effects models. We also evaluated the effects of ants on plant conditions (plant biomass of each plant group, total plant biomass and plant N contents of each plant group) and soil conditions (soil moisture, soil total available N and soil total available P) in the 3 × 3 m subplots in the six cattle grazed plots.

## 3. Results

### (a) Ant suppression success

Three years of ant suppression (2010–2012) led to greater than or equal to 96% reduction in total active ant nest densities, with 2.71 (s.e. 0.48) ant nests m^−2^ in ant-present subplots compared to 0.07 (s.e. 0.01) ant nests m^−2^ in the ant-suppressed subplots (*χ*^2^_1_ = 21.02, *p* < 0.001). Active nest densities of the dominant ant genus *Lasius* similarly dropped from 0.60 (s.e. 0.38) in the ant-present subplots to 0.02 (s.e. 0.02) in the ant-suppressed subplots (*χ*^2^_1_ = 20.15, *p* < 0.001).

### (b) Effects of cattle grazing on ants, plants, litter and microclimate

**Figure 2.**
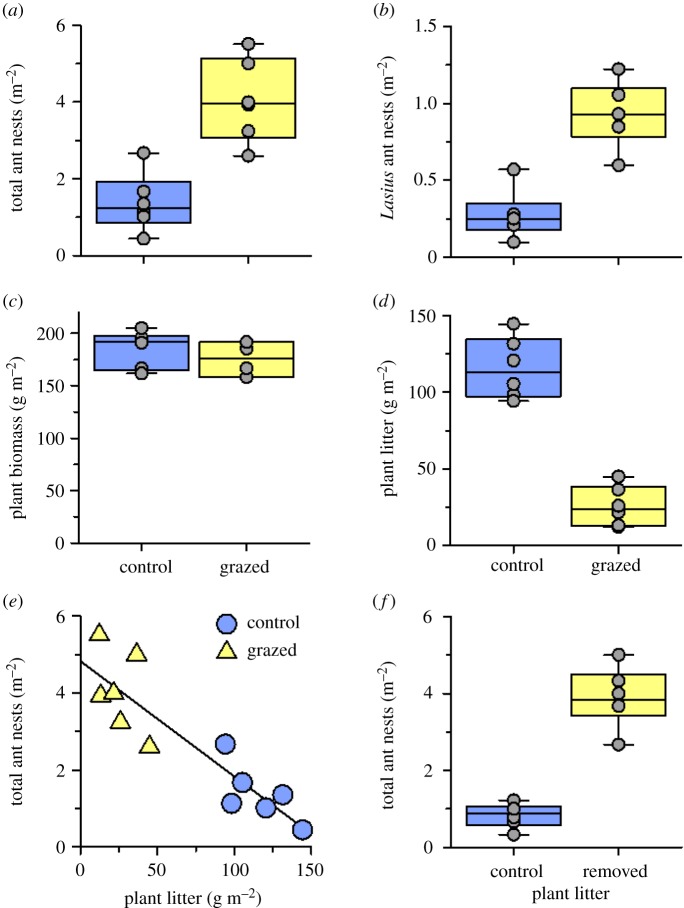
Effects of 3 yr (2010–2012) cattle grazing on (*a*) total ant nest density, (*b*) *Lasius* ant nest density, (*c*) total plant biomass, and (*d*) plant litter biomass in the ant-present subplots of the six control and grazed plots. (*e*) The effects of plant litter biomass on total ant nest density in the ant-present subplots of the control and grazed plots. (*f*) Total ant nest density in the plots where litter was either intact (control) or removed in the plant-litter-removal experiment in 2013. Presented are the median, the lower and upper quartiles at 25% and 75%, respectively, and the single values.

Three years of cattle grazing increased total active ant nest density by nearly twofold (*χ*^2^_1_ = 14.92, *p* = 0.001; figure 2*a*), and increased *Lasius* ant nest density threefold (*χ*^2^_1_ = 18.80, *p* < 0.001; figure 2*b*) in the ant-present (control) subplots. Cattle grazing did not significantly affect total plant biomass (*χ*^2^ = 1.27, *p* = 0.26; figure 2*c*), but grazing decreased plant litter biomass at the soil surface by 78% (*χ*^2^ = 29.73, *p* < 0.0001; figure 2*d*). Regression analyses showed that total ant nest density was negatively correlated with plant litter biomass (*R*^2^ = 0.79, *t*_1,5_ = −6.53, *p* < 0.001; figure 2*e*) in the ant-present subplots. Moreover, cattle grazing increased the percentage of light penetration at the soil surface in the ant-present subplots by 1.3-fold (*χ*^2^_1_ = 29.16, *p* < 0.001; electronic supplementary material, figure S2a), while air temperature and air relative humidity at the soil surface were not significantly affected (electronic supplementary material, figure S2b,c).

### (c) Effects of ants on cattle feeding behaviour, plants and soils

**Figure 3.**
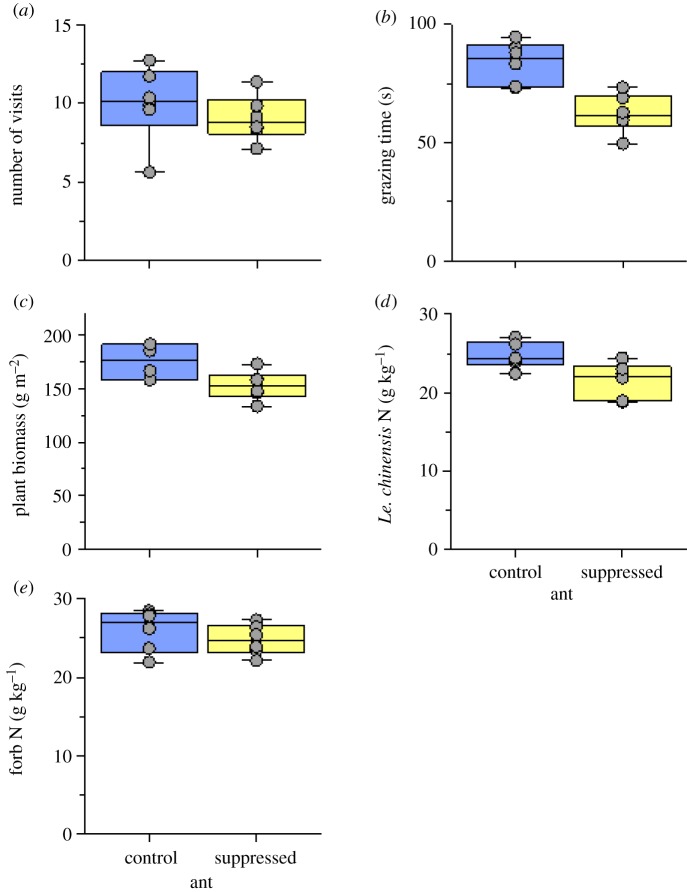
Effects of 3 yr (2010–2012) ant suppression on (*a*) total number of visits per subplot, (*b*) total grazing time per subplot by cattle, (*c*) total plant biomass, (*d*) *Le. chinensis* N content, and (*e*) forb N content in the 3 × 3 m treatment subplots in the six cattle grazed plots. Presented are the median, the lower and upper quartiles at 25% and 75%, respectively, and the single values.

The total number of cattle visits per subplot was not significantly affected by the suppression of ants in the grazed plots (*χ*^2^_1_ = 0.95, *p* = 0.33; figure 3*a*). However, the total cattle grazing time was 25% lower in the ant suppression subplots compared to the control subplots (*χ*^2^_1_ = 12.69, *p* = 0.001; figure 3*b*). Ant suppression reduced total plant biomass by 13% (*χ*^2^_1_ = 6.34, *p* = 0.012; figure 3*c*) and N content of the dominant *L. chinensis* grass in the subplots by 12% (*χ*^2^_1_ = 7.26, *p* = 0.007; figure 3*d*). Moreover, ant suppression significantly decreased the total availability of N in the soil of the subplots by 17% (*χ*^2^_1_ = 6.43, *p* = 0.011; electronic supplementary material, figure S3b), whereas it did not significantly affect soil moisture nor soil total P availability (electronic supplementary material, figure S3a,c).

### (d) Additional plant-litter-removal experiment

The total active ant nest density was nearly fivefold higher in the plots where plant litter was artificially removed (gls, *t*_1;10_ = 8.93, *p* < 0.001; figure 2*f*).

## 4. Discussion

Our experimental study demonstrates reciprocal facilitative interactions between two phylogenetic distant animal taxa. Cattle grazing increased total ant nest abundance, while ants facilitated the food intake of cattle during the peak of the growing season. These reciprocal facilitative interactions exemplify synergistic amelioration of habitat and improvement of resource (food) availability between very different animal taxa. Our results highlight that the study of interspecific interactions between phylogenetically different animal taxa and their potential reciprocal feedbacks, yields insights about species coexistence and the assembly of animal communities.

### (a) How large herbivores facilitate ants

Cattle acted as ecosystem engineers by decreasing the amount of plant litter at the ground surface, which we separately demonstrated benefits the abundance of soil ants (figure 2*f*). Our results are in line with earlier studies which indicate that large herbivores are often influential ecosystem engineers in terrestrial ecosystems [20]. Large herbivore activities, such as grazing, trampling and wallowing, are known to accelerate plant litter fragmentation and decomposition, which significantly reduces litter in grazed areas [21]. Given the dramatic increase of active ant nest density in the litter removal experiment, litter reduction appears to be the primary mechanism of how cattle facilitate ants.

*Lasius* spp. ants, the dominant group in our system, prefer bare ground with a dry and sunny microenvironment, and, generally, avoid nesting in habitats with thick vegetation and/or litter accumulation [46–48]. There are several possible reasons why *Lasius* ants tend to avoid these areas. Dense litter impacts the microclimate at the soil surface leading to unfavourable temperature regimes for ants and potentially reduces the ability of ants to regulate microclimate in their nests. By their mound building activities, ants regulate the microclimate (temperature, aeration and humidity) in their nests, not only for the benefit of their own eggs and larvae, but also to create optimal conditions for root lice with which some *Lasius* species (*Lasius flavus*) live in close association [39,58]. Other potential involved mechanisms as to why ants avoid dense litter areas for their nests may include avoidance of fungi infection to their eggs or larva, reduced effectiveness of anti-predator behaviour, or reduced search and transport possibilities for their food items [22]. These mechanisms are difficult to isolate and evaluate independently, and this was beyond the scope of our study. Nevertheless, it appears from this study that litter reduction via cattle grazing may facilitate habitat quality for ants.

### (b) The reciprocal effects of ants on large herbivores

Ants, in their turn, facilitated the feeding activities of cattle: cattle spent more time on grazing in areas with ants compared to ant suppression areas. This conclusion is based on a behavioural rather than the fitness response of cattle to ant activities here, owing to the difficulty of measuring cattle fitness within the short-time study period. However, there is evidence that foraging quantity is a good indicator of herbivores' performance [59–61]. The increases in cattle grazing time has probably to do with the activities of ants that led to the increased soil N availability and enhanced biomass production and quality (N content) of forage plants in the ant-present plots. Ants may increase soil fertility by foraging, excretion and nest-building activities that accelerate plant debris decomposition and thus increase N import and enhance nutrient cycling rates that benefit plant growth [49–51]. Indeed, in addition to food resources, cattle may be attracted to the ant-present subplots by some more cryptic mechanisms, such as altered plant community composition and simply the presence of ant mounds. For example, there is evidence that the presence of specific plant species or plant groups will modify the feeding preferences of herbivores on their hosts, a phenomenon called ‘plant associational effect’ [62–64]. The presence of ants increased the abundance of forb species in our system (X. Li, Z. Zhong, D. Wang, Y. Zhu, H. Zhu, L. Wang, N. Hassan 2018, unpublished data). Although the majority of cattle diet may be commonly carbon-rich grasses [53], there is also evidence that the search for N-rich forbs can be an important component to cattle foraging behaviours [65]. Thus, it is still unclear if, and to what degree, the increases in cattle grazing time in the ant-present sites can be attributed to the increases in forb abundance.

In our study, we found that the ants exerted a significant positive influence on a large mammal and vice versa. Although the latter dominates the literature [20,34–36], there is also growing evidence showing that smaller animals can exert effects on larger ones [37–41]. Our study adds to the list of such effects. In many ecosystems, invertebrates or small vertebrates—both above- and below-ground—often have as high as, or even higher, abundance or biomass compared with those of large vertebrates [66,67]. Given that all these animals often coexist within the same ecosystems and interact frequently, the potential reciprocal feedbacks of smaller animals on the larger ones are probably common and should not be ignored.

### (c) Phylogenetic distance and the balance of animal competition and facilitation

It is suggested that the phylogenetic or ecological distance (which are often correlated with each other [68]) among co-occurring organisms is a good proxy to predict the outcome of species interactions (i.e. competitive or facilitative) in natural communities [69,70]. This is rooted in the view that closely related organisms often have similar morphology and behaviour, require similar kinds of resources, and tend to compete for the same niche. Distantly related species, by contrast, may be more likely to coexist (or facilitate) because they exploit different niches. This hypothesis has been well documented in plant and microorganism communities [69,70], but much less in animal communities. Multiple studies have found that closely related herbivore species, such as sap-feeding insects [71] or livestock and wild ungulates [21] do tend to compete with each other. At the same time, a growing body of literature indicates the existence of interspecific facilitative interactions between a wide range of phylogenetic taxa, such as elephants and lizards [20], and beavers and waterbirds [23]. Our study adds to that body of literature. While this does not mean that competition between distantly related species, or facilitation between closely related species do not exist [38,45,72–76], it seems that in general, phylogenetic or ecological distance is a fairly good predictor for the competition–facilitation balance in animal communities, just as it is for plant communities [69,70]. However, the fact that there are many exceptions indicates that this relationship between phylogenetic distance and competition–facilitation balance in animal communities is a complex one. Currently, our understanding of the patterns and mechanisms of interspecific facilitation in animal communities still lags far behind our understanding of facilitation in plant communities. More studies are needed on the relationships between phylogenetic distance and the balance of competition and facilitation to improve our understanding of species coexistence and animal community assembly rules.

## Supplementary Material

Experimental design and the additional ant bait experiments

## Data accessibility

The data used for this study are available from the Dryad Digital Repository: http://dx.doi.org/10.5061/dryad.s7423sv [77].

## References

[1] Bertness MD, Callaway R 1994 Positive interactions in communities. Trends Ecol. Evol. 9, 191–193. (10.1016/0169-5347(94)90088-4) 21236818

[2] Callaway RM 1995 Positive interactions among plants. Bot. Rev. 61, 306–349. (10.1007/BF02912621)

[3] Stachowicz JJ 2001 Mutualism, facilitation, and the structure of ecological communities. BioScience 51, 235–246. (10.1641/0006-3568(2001)051%5B0235:MFATSO%5D2.0.CO;2)

[4] Brooker RW et al 2007 Facilitation in plant communities: the past, the present, and the future. J. Ecol. 96, 18–34. (10.1111/j.1365-2745.2007.01295.x)

[5] Bronstein JL 2009 The evolution of facilitation and mutualism. J. Ecol. 97, 1160–1170. (10.1111/j.1365-2745.2009.01566.x)

[6] He Q, Bertness MD 2014 Extreme stresses, niches, and positive species interactions along stress gradients. Ecology 95, 1437–1443. (10.1890/13-2226.1) 25039207

[7] Soliveres S, Smit C, Maestre FT 2015 Moving forward on facilitation research: response to changing environments and effects on the diversity, functioning and evolution of plant communities. Biol. Rev. 90, 297–313. (doi:10.1111%2Fbrv.12110) 24774563 10.1111/brv.12110 PMC4407973

[8] Bruno JF, Stachowicz JJ, Bertness MD 2003 Inclusion of facilitation into ecological theory. Trends Ecol. Evol. 18, 119–125. (10.1016/S0169-5347(02)00045-9)

[9] Sanders D, Jones CG, Thébault E, Bouma TJ, van der Heide T, van Belzen J, Barot S 2014 Integrating ecosystem engineering and food webs. Oikos 123, 513–524. (10.1111/j.1600-0706.2013.01011.x)

[10] Silliman BR, He Q 2018 Physical stress, consumer control, and new theory in ecology. Trends Ecol. Evol. 2392, 1–12. (10.1016/j.tree.2018.04.015) 29802026

[11] Callaway RM et al 2002 Positive interactions among alpine plants increase with stress. Nature 417, 844–888. (10.1038/nature00812) 12075350

[12] Maestre FT, Callaway RM, Valladares F, Lortie CJ 2009 Refining the stress-gradient hypothesis for competition and facilitation in plant communities. J. Ecol. 97, 199–205. (10.1111/j.1365-2745.2008.01476.x)

[13] Bertness MD, Leonard GH 1997 The role of positive interactions in communities: lessons from intertidal habitats. Ecology 78, 1976–1989. (10.1890/0012-9658(1997)078%5B1976:TROPII%5D2.0.CO;2)

[14] Smit C, Vandenberghe C, Den Ouden J, Müller-Schärer H 2007 Nurse plants, tree saplings and grazing pressure: changes in facilitation along a biotic environmental gradient. Oecologia 152, 265–273. (doi:0.1007/s00442-006-0650-6) 17279351 10.1007/s00442-006-0650-6

[15] Smit C, den Ouden J, Díaz M 2008 Facilitation of *Quercus ilex* recruitment by shrubs in Mediterranean open woodlands. J. Veg. Sci. 19, 193–200. (10.3170/2007-8-18352)

[16] Gross K 2008 Positive interactions among competitors can produce species-rich communities. Ecol. Lett. 11, 929–936. (10.1111/j.1461-0248.2008.01204.x) 18485001

[17] McIntire EJ, Fajardo A 2014 Facilitation as a ubiquitous driver of biodiversity. New Phytol. 201, 403–416. (10.1111/nph.12478) 24102266

[18] van der Wal R, van Wijnen H, van Wieren S, Beucher O, Bos D 2000 On facilitation between herbivores: how Brent Geese profit from brown hares. Ecology 81, 969–980. (10.1890/0012-9658(2000)081%5B0969:OFBHHB%5D2.0.CO;2)

[19] Arsenault R, Owen-Smith N 2002 Facilitation versus competition in grazing herbivore assemblages. Oikos 97, 313–318. (10.1034/j.1600-0706.2002.970301.x)

[20] Pringle RM 2008 Elephants as agents of habitat creation for small vertebrates at the patch scale. Ecology 89, 26–33. (10.1890/07-0776.1) 18376543

[21] Odadi WO, Karachi MK, Abdulrazak SA, Young TP 2011 African wild ungulates compete with or facilitate cattle depending on season. Science 333, 1753–1755. (10.1126/science.1208468) 21940896

[22] Karban R, Grof-Tisza P, Holyoak M 2012 Facilitation of tiger moths by outbreaking tussock moths that share the same host plants. J. Anim. Ecol. 81, 1095–1102. (10.1111/j.1365-2656.2012.01993.x) 22553976

[23] Nummi P, Holopainen S 2014 Whole-community facilitation by beaver: ecosystem engineer increases waterbird diversity. Aquat. Conserv. Freshw. Ecosyst. 24, 623–633. (10.1002/aqc.2437)

[24] Vázquez DP 2002 Multiple effects of introduced mammalian herbivores in a temperate forest. Biol. Invasions 4, 175–191. (10.1023/A:1020522923905)

[25] McNaughton SJ 1976 Serengeti migratory wildebeest: facilitation of energy flow by grazing. Science 191, 92–94. (10.1126/science.191.4222.92) 17834943

[26] Jones CG, Lawton JH, Shachak M 1994 Organisms as ecosystem engineers. Oikos 69, 373–386. (10.1007/978-1-4612-4018-1_14)

[27] Jones CG, Lawton JH, Shachak M 1997 Positive and negative effects of organisms as ecosystem engineers. Ecology 78, 1946–1957. (10.1890/0012-9658(1997)078%5B1946:PANEOO%5D2.0.CO;2)

[28] Wright JP, Jones CG, Flecker AS 2002 An ecosystem engineer, the beaver, increases species richness at the landscape scale. Oecologia 132, 96–101. (10.1007/s00442-002-0929-1) 28547281

[29] Bartel RA, Haddad NM, Wright JP 2010 Ecosystem engineers maintain a rare species of butterfly and increase plant diversity. Oikos 119, 883–890. (10.1111/j.1600-0706.2009.18080.x)

[30] Nummi P, Kattainen S, Ulander P, Hahtola A 2011 Bats benefit from beavers: a facilitative link between aquatic and terrestrial food webs. Biodivers. Conserv. 20, 851–859. (10.1007/s10531-010-9986-7)

[31] Holt RD, Lawton JH 1994 The ecological consequences of shared natural enemies. Annu. Rev. Ecol. Evol. Syst. 25, 495–520. (10.1146/annurev.es.25.110194.002431)

[32] Young TP, Palmer TM, Gadd ME 2005 Competition and compensation among cattle, zebras, and elephants in a semi-arid savanna in Laikipia, Kenya. Biol. Conserv. 122, 351–359. (10.1016/j.biocon.2004.08.007)

[33] Kimuyu DM, Veblen KE, Riginos C, Chira RM, Githaiga JM, Young TP 2017 Influence of cattle on browsing and grazing wildlife varies with rainfall and presence of megaherbivores. Ecol. Appl. 27, 786–798. (10.1002/eap.1482) 27935669

[34] Danell K, Huss-Danell K 1985 Feeding by insects and hares on birches earlier affected by moose browsing. Oikos 44, 75–81. (10.2307/3544046)

[35] Olofsson J, Strengbom J 2000 Response of galling invertebrates on *Salix lanata* to reindeer herbivory. Oikos 91, 493–498. (10.1034/j.1600-0706.2000.910310.x)

[36] Cease AJ, Elser JJ, Ford CF, Hao S, Kang L, Harrison JF 2012 Heavy livestock grazing promotes locust outbreaks by lowering plant nitrogen content. Science 335, 467–469. (10.1126/science.1214433) 22282812

[37] Palmer TM, Young TP, Stanton ML, Wenk E 2000 Short-term dynamics of an acacia ant community in Laikipia, Kenya. Oecologia 123, 425–435. (10.1007/s004420051030) 28308598

[38] Mobæk R, Narmo AK, Moe SR 2005 Termitaria are focal feeding sites for large ungulates in Lake Mburo National Park, Uganda. J. Zool. 267, 97–102. (10.1017/S0952836905007272)

[39] Veen GFC, Geuverink E, Olff H 2012 Large grazers modify effects of aboveground–belowground interactions on small-scale plant community composition. Oecologia 168, 511–518. (doi:0.1007/s00442-011-2093-y) 21863246 10.1007/s00442-011-2093-y PMC3261403

[40] Zhong ZW, Wang DL, Zhu H, Wang L, Feng C, Wang ZN 2014 Positive interactions between large herbivores and grasshoppers, and their consequences for grassland plant diversity. Ecology 95, 1055–1064. (10.1890/13-1079.1) 24933823

[41] Howison RA, Olff H, Koppel J, Smit C 2017 Biotically driven vegetation mosaics in grazing ecosystems: the battle between bioturbation and biocompaction. Ecol. Monogr. 87, 363–378. (10.1002/ecm.1259)

[42] Howison RA, Olff H, Steever R, Smit C 2015 Large herbivores change the direction of interactions within plant communities along a salt marsh stress gradient. J. Veg. Sci. 26, 1159–1170. (10.1111/jvs.12317)

[43] Levick SR, Asner GP, Kennedy-Bowdoin T, Knapp DE 2010 The spatial extent of termite influences on herbivore browsing in an African savanna. Biol. Conserv. 143, 2462–2467. (10.1016/j.biocon.2010.06.012)

[44] Veblen KE 2012 Savanna glade hotspots: plant community development and synergy with large herbivores. J. Arid Environ. 78, 119–127. (10.1016/j.jaridenv.2011.10.016)

[45] Wilcox TM, Schwartz MK, Lowe WH 2018 Evolutionary community ecology: time to think outside the (taxonomic) box. Trends Ecol. Evol. 33, 240–250. (10.1016/j.tree.2018.01.014) 29496340

[46] Hou JH, Zhou DW, Jiang SC 2002 Species composition and spatial distribution of ants in the grassland region in the west of Jilin province (in Chinese with English abstract). Acta Ecol. Sin. 22, 1781–1787.

[47] Holec M, Frouz J, Pokorný R 2006 The influence of different vegetation patches on the spatial distribution of nests and the epigeic activity of ants (*Lasius niger*) on a spoil dump after brown coal mining (Czech Republic). Eur. J. Soil Biol. 42, 158–165. (10.1016/j.ejsobi.2005.12.005)

[48] Dostál P 2007 Population dynamics of annuals in perennial grassland controlled by ants and environmental stochasticity. J. Veg. Sci. 18, 91–102. (10.1111/j.1654-1103.2007.tb02519.x)

[49] Wu HT, Batzer DP, Yan XM, Lu XG, Wu DH 2013 Contributions of ant mounds to soil carbon and nitrogen pools in a marsh wetland of Northeastern China. Appl. Soil Ecol. 70, 9–15. (10.1016/j.apsoil.2013.04.004)

[50] Ehrle A, Andersen AN, Levick SR, Schumacher J, Trumbore SE, Michalzik B 2017 Yellow-meadow ant (*Lasius flavus*) mound development determines soil properties and growth responses of different plant functional types. Eur. J. Soil Biol. 81, 83–93. (10.1016/j.ejsobi.2017.06.006.)

[51] Farji-Brener AG, Werenkraut V 2017 The effects of ant nests on soil fertility and plant performance: a meta-analysis. J. Anim. Ecol. 86, 866–877. (10.1111/1365-2656.12672) 28369906

[52] Blomqvist MM, Olff H, Blaauw MB, Bongers T, Van der Putten WH 2000 Interactions between above- and belowground biota: importance for small-scale vegetation mosaics in a grassland ecosystem. Oikos 90, 582–598. (10.1034/j.1600-0706.2000.900316.x)

[53] Liu J, Feng C, Wang DL, Wang L, Wilsey BJ, Zhong ZW 2015 Impacts of grazing by different large herbivores in grassland depend on plant species diversity. J. Appl. Ecol. 52, 1053–1062. (10.1111/1365-2664.12456)

[54] Li XF, Liu JS, Fan J, Ma YN, Ding SW, Zhong ZW, Wang D 2015 Combined effects of nitrogen addition and litter manipulation on nutrient resorption of *Leymus chinensis* in a semi-arid grassland of northern China. Plant Biol. 17, 9–15. (10.1111/plb.12172) 24666511

[55] Wardle DA, Hyodo F, Bardgett RD, Yeates GW, Nilsson MC 2011 Long-term aboveground and belowground consequences of red wood ant exclusion in boreal forest. Ecology 92, 645–656. (10.1890/10-1223.1) 21608473

[56] R Development Core Team. 2014 R: a language and environment for statistical computing. Vienna, Austria: R Foundation for Statistical Computing.

[57] Pinheiro J, Bates D, DebRoy S, Sarkar D 2014 R Core Team (2014) nlme: linear and nonlinear mixed effects models. R package version 3.1–117. See http://CRAN.R-project.org/package=nlme.

[58] Sanders D, Platner C 2007 Intraguild interactions between spiders and ants and top-down control in a grassland food web. Oecologia 150, 611–624. (10.1007/s00442-006-0538-5) 17091284

[59] Bailey DW, Gross JE, Laca EA, Rittenhouse LR, Coughenour MB, Swift DM, Sims PL 1996 Mechanisms that result in large herbivore grazing distribution patterns. J. Range Manag. 49, 386–400. (10.2307/4002919)

[60] Van Beest FM, Mysterud A, Loe LE, Milner JM 2010 Forage quantity, quality and depletion as scale-dependent mechanisms driving habitat selection of a large browsing herbivore. J. Anim. Ecol. 79, 910–922. (10.1111/j.1365-2656.2010.01701.x) 20443990

[61] Wang L, Wang DL, He ZB, Liu GF, Hodgkinson KC 2010 Mechanisms linking plant species richness to foraging of a large herbivore. J. Appl. Ecol. 47, 868–875. (10.1111/j.1365-2664.2010.01837.x)

[62] Root RB 1973 Organization of a plant-arthropod association in simple and diverse habitats: the fauna of collards (*Brassica oleracea*). Ecol. Monogr. 43, 95–124. (10.2307/1942161)

[63] Barbosa P, Hines J, Kaplan I, Martinson H, Szczepaniec A, Szendrei Z 2009 Associational resistance and associational susceptibility: having right or wrong neighbors. Annu. Rev. Ecol. Evol. Syst. 40, 1–20. (10.1146/annurev.ecolsys.110308.120242)

[64] Underwood N, Inouye BD, Hambäck PA 2014 A conceptual framework for associational effects: when do neighbors matter and how would we know? Quar. Rev. Biol. 89, 1–19. (10.1086/674991) 24672901

[65] Odadi WO, Abdulrazak SA, Karachi MM, Young TP 2013 Protein supplementation driven shifts in forage selection by cattle: implications for cattle wildlife coexistence. Ecol. Appl. 23, 455–463. (10.1890/12-0878.1) 23634594

[66] Wilson EO 1987 The little things that run the world (the importance and conservation of invertebrates). Conserv. Biol. 1, 344–346. (10.1111/j.1523-1739.1987.tb00055.x)

[67] Samson F, Knopf F 1994 Prairie conservation in North America. BioScience 44, 418–421. (10.2307/1312365)

[68] Webb CO, Ackerly DD, McPeek MA, Donoghue MJ 2002 Phylogenies and community ecology. Annu. Rev. Ecol. Evol. Syst. 33, 475–505. (10.1146/annurev.ecolsys.33.010802.150448)

[69] Valiente-Banuet A, Verdú M 2008 Temporal shifts from facilitation to competition occur between closely related taxa. J. Ecol. 96, 489–494. (10.1111/j.1365-2745.2008.01357.x)

[70] Mayfield MM, Levine JM 2010 Opposing effects of competitive exclusion on the phylogenetic structure of communities. Ecol. Lett. 13, 1085–1093. (10.1111/j.1461-0248.2010.01509.x) 20576030

[71] Denno RF, Peterson MA, Gratton C, Cheng J, Langellotto GA, Huberty AF, Finke DL 2000 Feeding-induced changes in plant quality mediate interspecific competition between sap-feeding herbivores. Ecology 81, 1814–1827. (10.1890/0012-9658(2000)081%5B1814:FICIPQ%5D2.0.CO;2)

[72] Huntzinger M, Karban R, Cushman JH 2008 Negative effects of vertebrate herbivores on invertebrates in a coastal dune community. Ecology 89, 1972–1980. (10.1890/07-0834.1) 18705383

[73] Bakker ES, Dobrescu I, Straile D, Holmgren M 2013 Testing the stress gradient hypothesis in herbivore communities: facilitation peaks at intermediate nutrient levels. Ecology 94, 1776–1784. (10.1890/12-1175.1) 24015521

[74] Gordon IJ 1988 Facilitation of red deer grazing by cattle and its impact on red deer performance. J. Appl. Ecol. 25, 1–9. (10.2307/2403605)

[75] Ohgushi T 2005 Indirect interaction webs: herbivore-induced effects through trait change in plants. Annu. Rev. Ecol. Evol. Syst. 36, 80–105. (10.1146/annurev.ecolsys.36.091704.175523)

[76] Xi X, Griffin JN, Sun S 2013 Grasshoppers amensalistically suppress caterpillar performance and enhance plant biomass in an alpine meadow. Oikos 122, 1049–1057. (10.1111/j.1600-0706.2012.00126.x)

[77] Li X et al. 2018 Data from: Reciprocal facilitation between large herbivores and ants in a semi-arid grassland *Dryad Digital Repository*. (10.5061/dryad.s7423sv) PMC6191696 30305439

