# Supplementary material for: Reciprocal facilitation between large herbivores and ants in a semi-arid grassland
Source: Proc Biol Sci. 2018 Oct 10;285(1888):20181665. doi: 10.1098/rspb.2018.1665 (PMC6191696; doi:10.1098/rspb.2018.1665)
Supplement: Experimental design and the additional ant bait experiments [file rspb20181665supp1.doc]

**Supplementary Materials**

**Running Head:** Facilitation between cattle and ants

**Reciprocal facilitation between large herbivores and ants in a semi-arid grassland**

Xiaofei Li^1^, Zhiwei Zhong^1*^, Dirk Sanders^2^, Christian Smit^3^, Deli Wang^1*^, Petri Nummi^4^, Yu Zhu^1^, Ling Wang^1^, Hui Zhu^1^, Nazim Hassan^1^

*^1^Institute of Grassland Science/School of Environment, Northeast Normal University, and Key Laboratory of Vegetation Ecology/Key Laboratory for Wetland Ecology and Vegetation Restoration, Changchun, Jilin 130024, China*

*^2^Environment and Sustainability Institute, University of Exeter, Penryn Campus, Penryn, Cornwall, TR10 9FE, United Kingdom*

*^3^Conservation Ecology Group, Groningen Institute for Evolutionary Life Sciences, University of Groningen, P.O. Box 11103, 9700 CC Groningen, The Netherlands*

*^4^Wetland Ecology Group, Department of Forest Sciences, University of Helsinki, P.O.Box 27, FI-00014 University of Helsinki, Finland*

Corresponding author: Deli Wang, Email: [wangd@nenu.edu.cn](mailto:wangd@nenu.edu.cn); Zhiwei Zhong, Email: [zhongzw822@nenu.edu.cn](mailto:zhongzw822@nenu.edu.cn)

Article DOI: 10.1098/rspb.2018.1665

**Supplementary Materials**

**Figure S1** Figure of the experimental layout of this study.


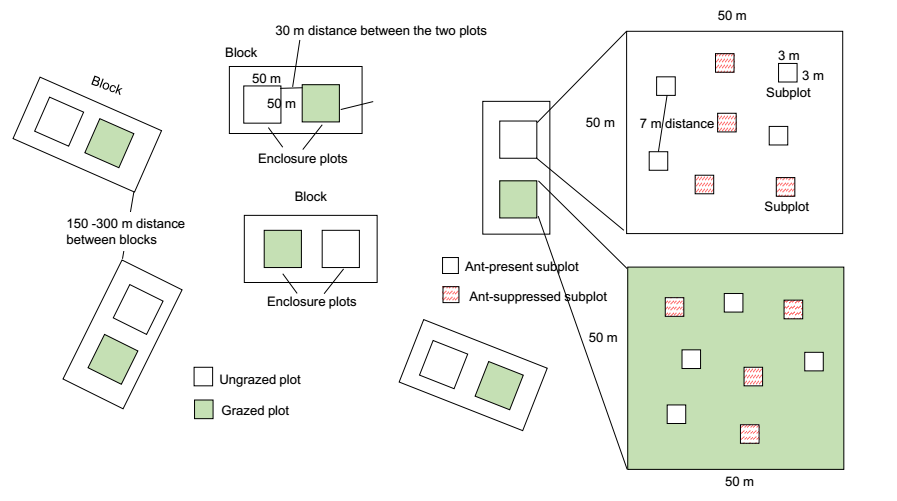


**Figure S2** Effects of 3-yr (2010-2012) cattle grazing on (a) percentage of light penetrate to soil surface, (b) air temperature, and (c) air relative humidity at soil surface in the ant-present subplots of the six control and grazed plots. Presented are the median, the lower and upper quartiles at 25% and 75%, and the single values.


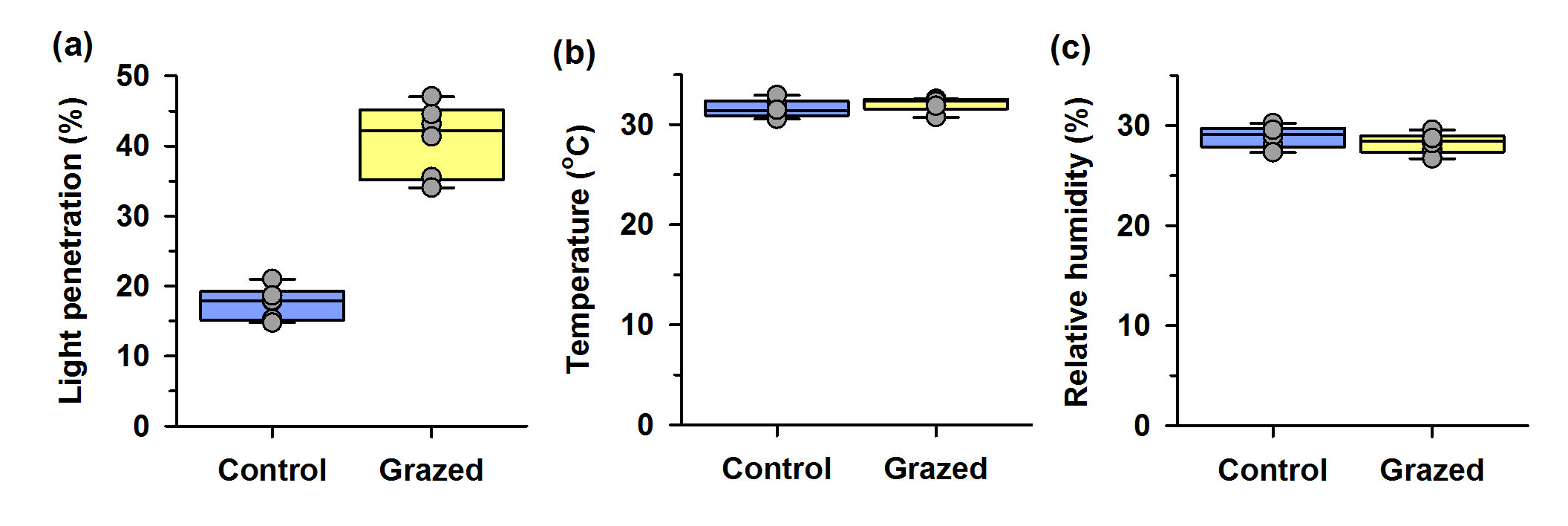


**Figure S3** Effects of 3-yr (2010-2012) ant suppression on (a) soil moisture, (b) soil available nitrogen (N), and (c) soil available phosphorus (P) in the 3 × 3 m treatment subplots in the six cattle grazed plots. Presented are the median, the lower and upper quartiles at 25% and 75%, and the single values.


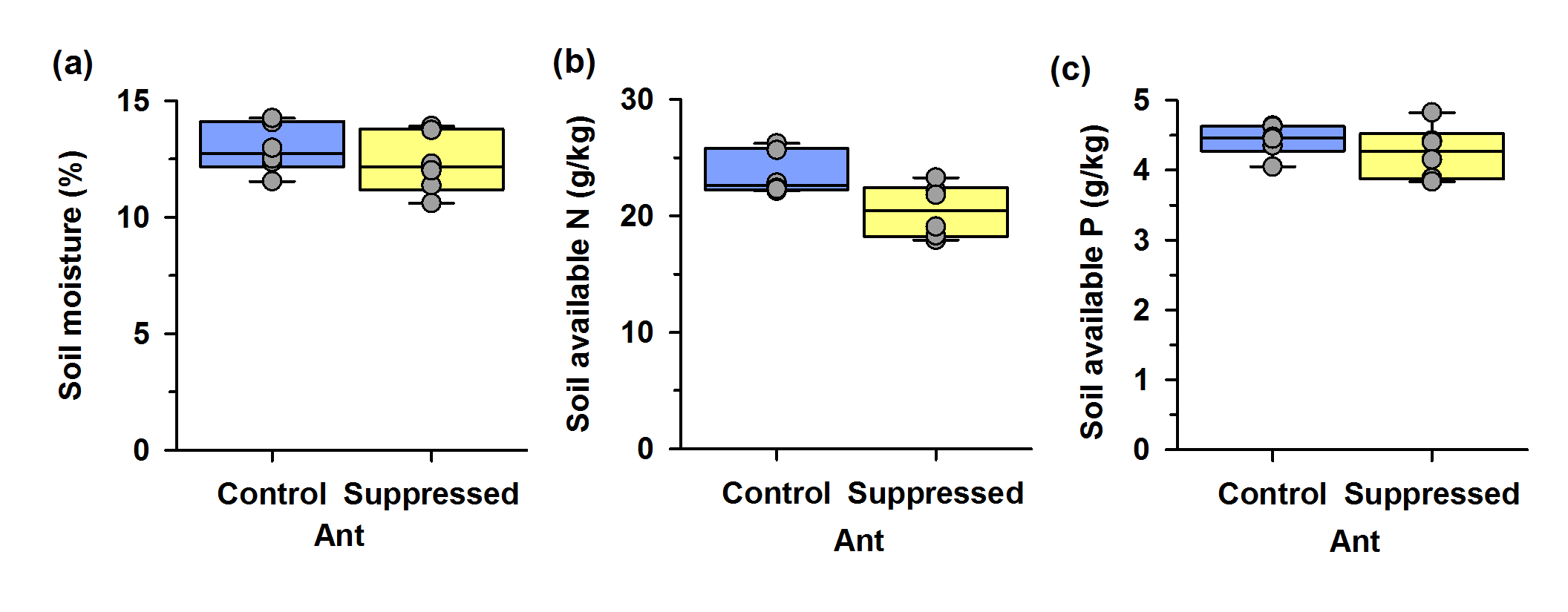


**Table S1.** The initial plant biomass of the three plant groups (the dominant *L. chinensis* grasses, other grasses, and forbs), total plant biomass, total plant litter biomass, total ant nest density, and *Lasius* ant nest density in the four treatment subplots (ants only – “A”, no cattle and ants – “N”, cattle and ants – “C + A”, and cattle only – “C”) in August 2009, one year before the beginning of cattle grazing, in the large-scale field grazing experiments.

|  | Treatment | | | |  |  |
| --- | --- | --- | --- | --- | --- | --- |
| Variable | A | N | C + A | C | *F* | *P* |
| *L. chinensis* biomass (g m^-2^) | 123.39 (10.46) | 130.38 (10.39) | 131.19 (7.68) | 126.17 (8.66) | 0.92 | 0.45 |
| Other grass biomass (g m^-2^) | 23.93 (3.23) | 22.02 (3.65) | 25.49 (2.70) | 24.13 (2.37) | 1.33 | 0.29 |
| Forb biomass (g m^-2^) | 32.99 (4.00) | 32.93 (2.54) | 31.17 (3.93) | 32.26 (3.61) | 0.34 | 0.80 |
| Total plant biomass (g m^-2^) | 180.31 (15.92) | 185.33 (8.25) | 187.85 (7.99) | 182.56 (9.57) | 0.54 | 0.66 |
| Total litter biomass (g m^-2^) | 132.81 (13.39) | 127.78 (11.33) | 122.02 (16.56) | 129.55 (12.47) | 0.67 | 0.58 |
|  |  |  |  |  |  |  |
| Total ant nest density (no. m^-2^) | 1.26 (0.61) | 1.48 (0.25) | 1.42 (0.75) | 1.33 (0.40) | 0.20 | 0.89 |
| *Lasius* ant nest density (no. m^-2^) | 0.19 (0.09) | 0.26 (0.08) | 0.23 (0.13) | 0.19 (0.96) | 0.90 | 0.46 |

*Notes*: Data are means with SE in parentheses. *F* and *P* values are derived from one-way ANOVA with df = 3, 15. Values in boldface indicate a statistically significant effect of experimental treatment (*P* ≤ 0.05).

**Table S2.** The initial plant nutrient (nitrogen) contents of the three plant groups (the dominant *L. chinensis* grasses, other grasses, and forbs) in the four treatment subplots (ants only – “A”, no cattle and ants – “N”, cattle and ants – “C + A”, and cattle only – “C”) in August 2009, one year before the beginning of cattle grazing, in the large-scale field grazing experiments.

|  | Treatment | | | |  |  |
| --- | --- | --- | --- | --- | --- | --- |
| Nitrogen content (g kg^-1^) | A | N | C + A | C | *F* | *P* |
| *L. chinensis* grasses | 18.63 (1.48) | 18.36 (1.69) | 18.02 (1.29) | 19.05 (1.73) | 0.47 | 0.71 |
| Other grasses | 12.87 (0.88) | 13.69 (0.84) | 14.08 (1.17) | 13.19 (0.63) | 2.10 | 0.13 |
| Forbs | 23.62 (1.31) | 23.03 (2.14) | 22.75 (2.11) | 23.45 (1.52) | 0.29 | 0.84 |

*Notes*: Data are means with SE in parentheses. *F* and *P* values are derived from one-way ANOVA with df = 3, 15. Values in boldface indicate a statistically significant effect of experimental treatment (*P* ≤ 0.05).

***Table S3.*** *The initial soil moisture, soil available nitrogen (N), soil available phosphorus (P), light penetration, air temperature, and air relative humidity at soil surface in the four treatment subplots (ants only – “A”, no cattle and ants – “N”, cattle and ants – “C + A”, and cattle only – “C”) in August 2009, one year before the beginning of cattle grazing, in the large-scale field grazing experiments.*

|  | Treatment | | | |  |  |
| --- | --- | --- | --- | --- | --- | --- |
| Variable | A | N | C + A | C | *F* | *P* |
| Soil moisture (%) | 12.24 (0.83) | 12.45 (1.05) | 12.55 (0.85) | 12.38 (0.49) | 0.15 | 0.93 |
| Soil available N (mg kg^-1^) | 18.63 (1.47) | 18.16 (1.53) | 17.81 (2.08) | 18.83 (1.94) | 0.41 | 0.75 |
| Soil available P (mg kg^-1^) | 4.16 (0.38) | 3.81 (0.47) | 3.98 (0.37) | 4.08 (0.30) | 0.91 | 0.46 |
|  |  |  |  |  |  |  |
| Light penetration (%) | 15.11 (2.50) | 14.35 (1.52) | 15.34 (2.02) | 14.75 (1.86) | 0.52 | 0.67 |
| Air temperature (^o^C) | 30.99 (1.16) | 30.82 (0.87) | 30.58 (1.04) | 30.55 (1.09) | 0.24 | 0.87 |
| Air relative humidity (%) | 26.04 (1.61) | 25.88 (0.92) | 26.54 (1.24) | 26.11 (1.16) | 0.30 | 0.82 |

*Notes*: Data are means with SE in parentheses. *F* and *P* values are derived from one-way ANOVA with blocking and with df = 3, 15. Values in boldface indicate a statistically significant effect of experimental treatment (*P* ≤ 0.05).

**Additional experiments to examine the potential side effects of ant baits**

From 2012 to 2013, we conducted a set of additional experiments to test if Jingkang Ant Bait has side effects on ground living arthropods, plant living arthropods, plant growth, soil nutrient availability, and cattle behaviors in the system.

***Impacts of Jingkang Ant Bait on ground living and plant living arthropods***

In May 2012, six pairs of 3 × 3 m plots were placed in the field outside the grazing areas. We randomly selected one plot of each pair to serve as ant-suppression treatment, while the other plot served as the control. For the ant suppression plots, we applied 10 g of Jingkang Ant Bait Granules around the entrance of active ant nests to suppress ants from June to August of each year. We repeated the experimental treatments in the plots in 2013.

In mid-August 2013, we assessed the influences of ant bait applications on dominant ground living arthropods, including ants, beetles, crickets, and spiders, and dominant plant living arthropods, including grasshoppers, beetles, leafhoppers, and spiders. We sampled arthropod abundance in two dates, namely August 10 and 25. For ant abundance, we visually counted the total number of ant nests to present ant abundance in each plot. We used four 0.05 m^2^ pit-fall traps that located randomly within each plot to assess the abundance of other ground living arthropod in the plots. Pitfall traps were filled with propylene glycol (30 %) to preserve the arthropods [1]. Each sampling period traps were open for five consecutive days. We pooled the arthropods of the four pit-fall traps to present ground arthropod abundance in each plot. We used the standard sweep net survey method (0.5 m in diameter) to estimate plant living arthropod abundance in the plot [2, 3]. Five sweeps were taken within each plot. We pooled the arthropods of the five sweeps to present plant living arthropods abundance in each plot. We averaged ground living and plant living arthropod data from the two sampling dates in each plot, and then used them in the statistical analyses, respectively.

All data were assessed for normality and analyzed using the open source software R 3.1.0. The effects of ant bait application (two levels: control, ant bait application) on ants were analysed using linear models based on generalized least squares (with VarIdent) to account for unequal variances for the treatment groups, while other ground living arthropods (beetles, crickets, and spiders) and plant living arthropods (grasshoppers, beetles, leafhoppers, and spiders) were analysed using analysis of variance.

We found that the applications of ant bait decreased the abundance of total ant nests by nearly 17-fold (gls, *t*_1,10_ = 26.45, *P* < 0.001; Fig. S4a), but it increased cricket abundance by three-fold (*F*_1,10_ = 60.45, *P* < 0.001; Fig. S4c) in the plots. Ant bait applications have no impacts on other ground living arthropods and plant living arthropods in the plots.

**Figure S4.** Effects of 2-yr (2012-2013) Jingkang Ant Bait applications on the abundance of major ground living arthropods, including (a) ants, (b) beetles, (c) crickets, and (d) spiders, and the abundance of plant living arthropods, including (e) grasshoppers, (f) beetles, (g) leafhoppers, and (h) spiders in the plots. Presented are the median, the lower and upper quartiles at 25% and 75%, and the single values.


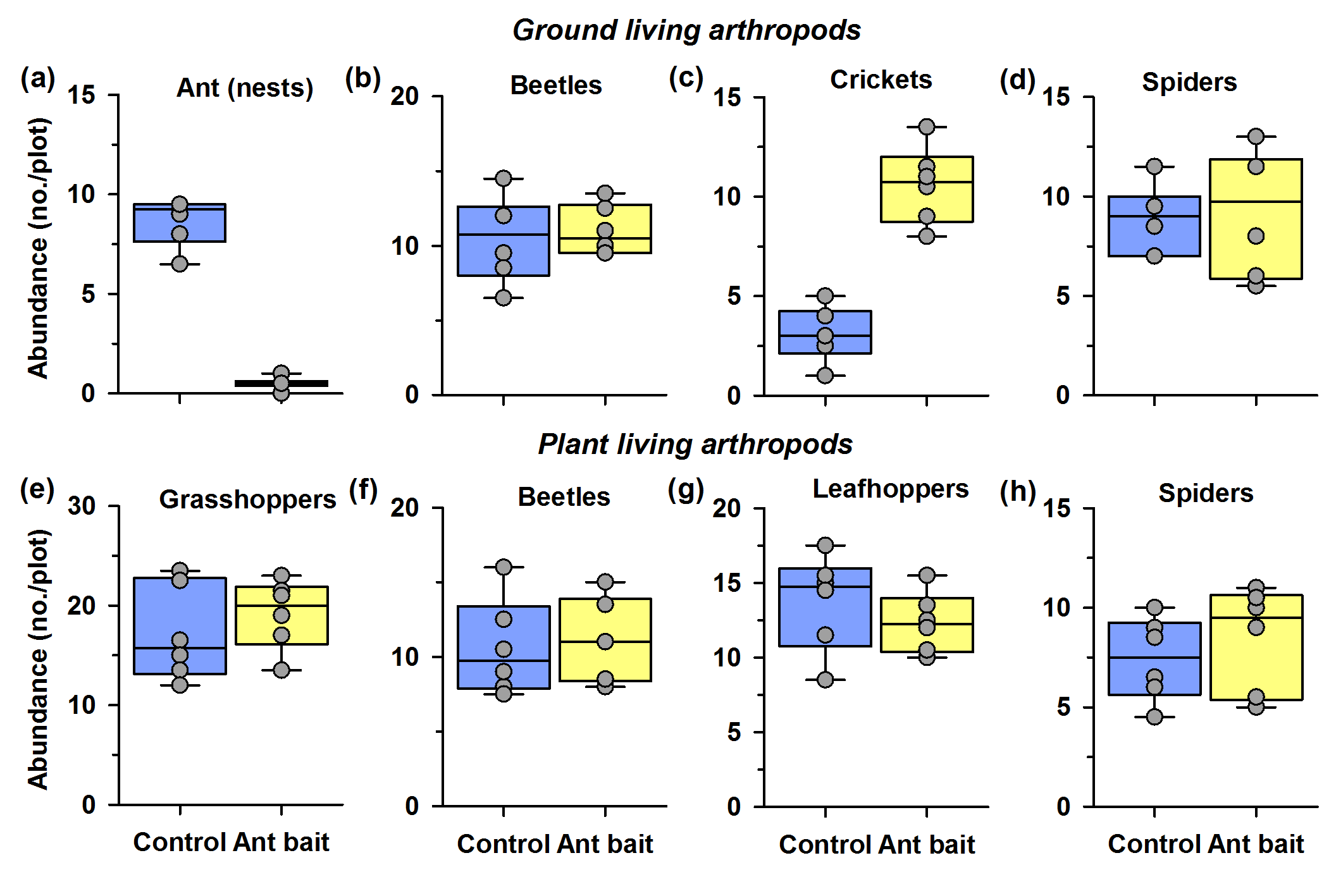


***Impacts of Jingkang Ant Bait on plant growth and soil nutrient availability***

During growing season (May-August) of 2012, we assessed if the Jingkang Ant Bait itself has side-effects on plant growth and soil nutrients. In May, we transplanted natural plant communities with soils from ungrazed sites into 20 flowerpots. The flowerpots are with 0.5 m^2^ base area and 0.3 m height. We put these flowerpots in the open spaces to receive ambient sunlight and rainfall. We checked the flowerpots weekly and removed all ground living and plant living arthropods by hands. We randomly selected 10 flowerpots to receive ant bait applications, whereas the others server as control. For the ant bait application treatments, we applied 0.56 g Jingkang Ant Bait Granules (the mean average dose we used in ant-suppression treatments in the large-scale grazing experiments) to the 10 flowerpots each month from June to August.

In August 25, we measured living plant biomass using a destructive method. We harvested plants in all the flowerpots by clipping plant tissue 2 cm above soil surface. Plants were sorted by grasses and forbs, then dried for 48 h at 70 °C and weighed.

Before plant harvest in August 25, we measured soil moisture in the flowerpots by taking reading from two random locations within each flowerpot using a handheld soil moisture reader (OSA-1, OUSU Technology, Hebei, China). Using a 4-cm-diameter soil auger, we randomly collected two replicate 0 – 20 cm soil samples from each flowerpot and then pooled these to homogenize the samples. For each soil sample, a 10 g subsample was extracted with 70 mL 2 mol L^-1^ KCl. Extracts were frozen at 20 °C for analysis of NH_4_^+^and NO_3_^-^ content by continuous flow analyzer (Alliance Flow Analyzer; Futura, Frépillon, France). Soil total available N concentration was the sum of NH_4_^+^and NO_3_^-^ concentrations. For soil total available P, another 10 g subsample soil was extracted using acidified NH4OAc-EDTA and analyzed by ICP (Spectro Analytical Instruments, Marlborough, MA, USA).

All data were assessed for normality, and if needed, normalized by log transformations and analyzed using the open source software R 3.1.0. The effects of ant bait application (two levels: control, ant bait application) on total plant biomass, grass biomass, forb biomass, soil moisture, soil available N, and soil available P were analysed using analysis of variance.

We found that the applications of ant bait did not affect total plant biomass (*F*_1,18_ = 0.29, *P* = 0.60; Fig. S5a), grass biomass (*F*_1,18_ = 0.98, *P* = 0.34; Fig. S5b), forb biomass (*F*_1,18_ = 0.30, *P* = 0.59; Fig. S5c), soil moisture (*F*_1,18_ = 0.02, *P* = 0.90; Fig. S5d), soil available N (*F*_1,18_ = 0.27, *P* = 0.61; Fig. S5e), and soil available P (*F*_1,18_ = 0.21, *P* = 0.65; Fig. S5f) in the flowerpots.

**Figure S5.** Effects of 1-yr (2012) Jingkang Ant Bait applications on (a) total plant biomass, (b) grass biomass, (c) soil moisture, (d) soil available N, and (e) soil available P in the flowerpots. Presented are the median, the lower and upper quartiles at 25% and 75%, and the single values.


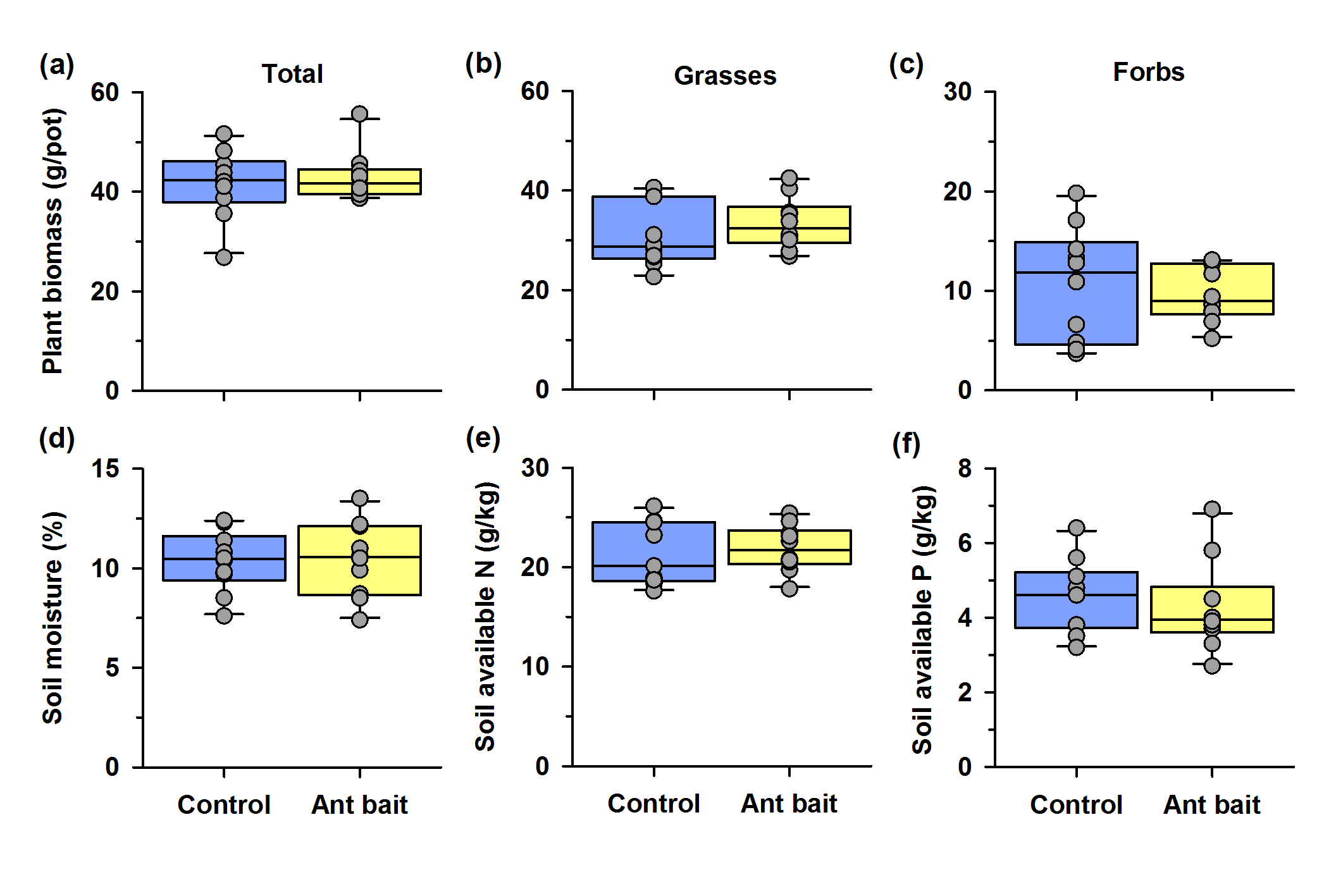


***Impacts of Jingkang Ant Bait on cattle behaviors***

In 2012, we assessed if the Jingkang Ant Bait itself has side effects on cattle behaviors. In May, we transplanted natural plant communities with soils from ungrazed sites into 40 flowerpots. The flowerpots are with 0.5 m^2^ base area and 0.3 m height. We put these flowerpots in the open spaces to receive ambient sunlight and rainfall. We checked the flowerpots weekly and removed all ground living and plant living arthropods by hands. We randomly selected 20 flowerpots to receive ant bait applications, whereas the others served as control. For the ant bait application treatment, we applied 0.56 g Jingkang Ant Bait Granules (the mean average dose we used in ant-suppression treatments in the large-scale grazing experiments) to the 20 flowerpots each month from June to August.

In August 23, we established a circle exclosure with diameter of 10 m (total area = 78.5 m^2^) in a bare land. We randomly assigned the 20 control and 20 ant-bait-application flowerpots into the exclosure. The distance between flowerpots is about 1 m. Then we randomly selected three cattle from the grazing sites, and allowed these cattle to do self-help feeding for 20 minutes in the exclosure. We used a hand-held computer to record the total number of visits and total grazing time (recorded and calculated to the second) by cattle of each flowerpots.

All data were assessed for normality and analyzed using the open source software R 3.1.0. The effects of ant bait application (two levels: control, ant bait application) on cattle total number of visits and total grazing time (second) to the flowerpots were analysed using analysis of variance.

We found that the applications of ant bait did not affect cattle total number of visits (*F*_1,38_ = 0.04, *P* = 0.85; Fig. S6a) and total grazing time (*F*_1,38_ = 0.01, *P* = 0.93; Fig. S6b) to the flowerpots.

**Figure S6.** Effects of 1-yr (2012) Jingkang Ant Bait applications on cattle (a) total number of visits, and (b) total grazing time (second) to the flowerpots. Presented are the median, the lower and upper quartiles at 25% and 75%, and the single values.


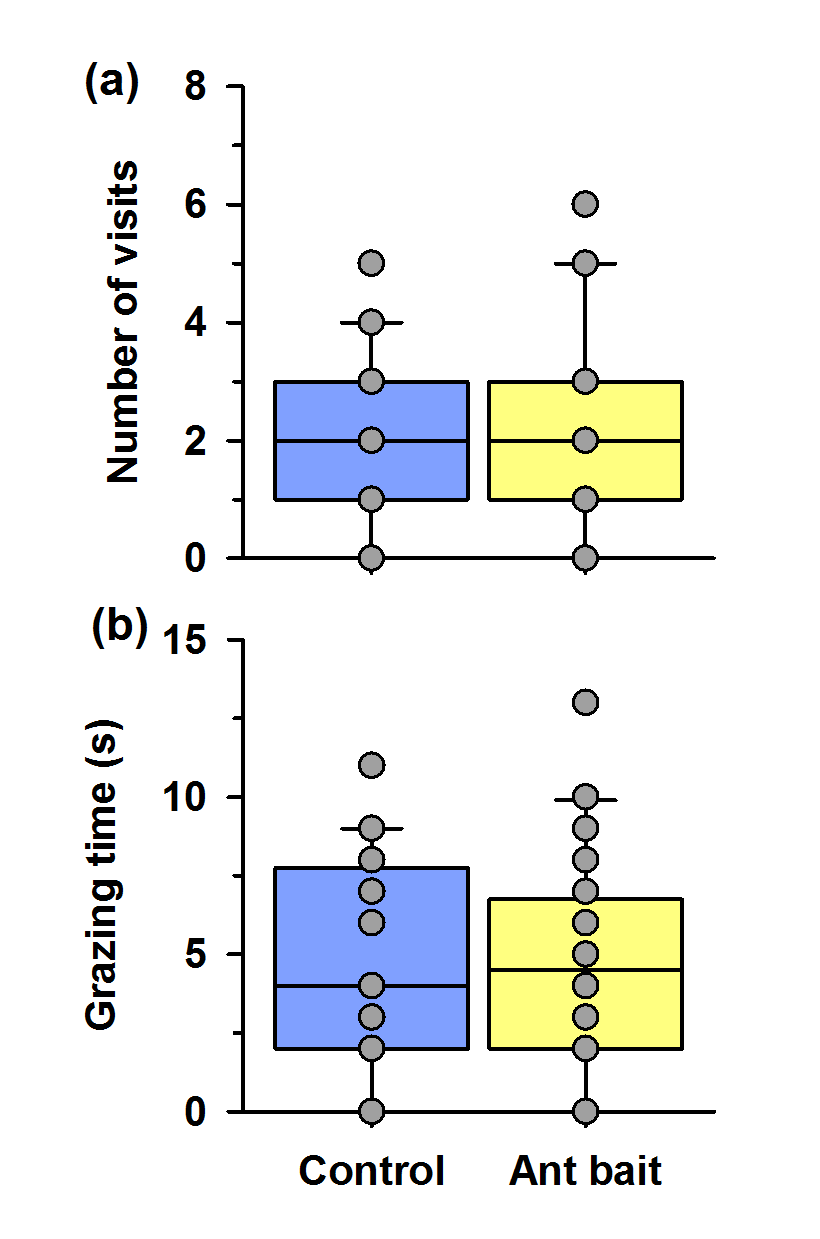


**References:**

1. Audino LD, Louzada J Comita L. 2014 Dung beetles as indicators of tropical forest restoration success: is it possible to recover species and functional diversity? *Biol. Conserv.* 169, 248–257. (doi: 10.1016/j.biocon.2013.11.023)

2. Haddad NM, Tilman D, Haarstad J, Ritchie ME, Knops JMH. 2001 Contrasting effects of plant richness and composition on insect communities: a field experiment. *Am. Nat.* 158, 17–35. (doi: 10.1086/320866)

3. Joern A. 2005 Disturbance by fire frequency and bison grazing modulate

grasshopper assemblages in tallgrass prairie. *Ecology* 86, 861–873. (doi: 10.1890/04-0135)
